# In silico biological discovery with large perturbation models

**DOI:** 10.1038/s43588-025-00870-1

**Published:** 2025-10-15

**Authors:** Djordje Miladinovic, Tobias Höppe, Mathieu Chevalley, Andreas Georgiou, Lachlan Stuart, Arash Mehrjou, Marcus Bantscheff, Bernhard Schölkopf, Patrick Schwab

**Affiliations:** 1 GSK plc, Zug, Switzerland; 2 Helmholtz Munich, Tübingen, Germany; 3 Max Planck Institute for Intelligent Systems, Tübingen, Germany; 4 ELLIS Institute, Tübingen, Germany

**Keywords:** Computational biology and bioinformatics, Drug screening

## Abstract

Data generated in perturbation experiments link perturbations to the changes they elicit and therefore contain information relevant to numerous biological discovery tasks—from understanding the relationships between biological entities to developing therapeutics. However, these data encompass diverse perturbations and readouts, and the complex dependence of experimental outcomes on their biological context makes it challenging to integrate insights across experiments. Here we present the large perturbation model (LPM), a deep-learning model that integrates multiple, heterogeneous perturbation experiments by representing perturbation, readout and context as disentangled dimensions. LPM outperforms existing methods across multiple biological discovery tasks, including in predicting post-perturbation transcriptomes of unseen experiments, identifying shared molecular mechanisms of action between chemical and genetic perturbations, and facilitating the inference of gene–gene interaction networks. LPM learns meaningful joint representations of perturbations, readouts and contexts, enables the study of biological relationships in silico and could considerably accelerate the derivation of insights from pooled perturbation experiments.

## Abstract (web summary)

A large perturbation model that integrates diverse laboratory experiments is presented to predict biological responses to chemical or genetic perturbations and support various biological discovery tasks.

## Main

Perturbation experiments play a central role in elucidating the underlying causal mechanisms that govern the behaviors of biological systems^1–3^. Controlled perturbation experiments measure changes in experimental readouts, such as the number of specific transcripts observed, resulting from introducing perturbations to biological systems, such as in vitro cell lines, compared with unperturbed references. Researchers use controlled perturbations in relevant biological model systems to establish causal relationships between molecular mechanisms, genes, chemical compounds and disease phenotypes. This causal understanding of foundational biological relationships has the potential to positively impact numerous important societal goals^4^, including the production of climate-friendly foods and materials and the development of novel therapeutics that address unmet health needs.

The path to understanding complex biological systems and developing targeted therapeutics hinges on unraveling how cells respond to perturbations. High-throughput experiments have generated an unprecedented volume of perturbation data spanning thousands of perturbations across diverse readout modalities and biological contexts, from single-cell to in vivo settings^5–9^. However, these experiments, while rich in indispensable information, vary dramatically in their protocols, readouts and model systems, often with minimal overlap. The vast scale and heterogeneity of this data, compounded by context-specific effects, make it extremely challenging to derive generalizable biological insights that drive scientific discovery. A core challenge in integrating evidence collected across heterogenous experiments is that it is difficult to disentangle effects stemming from differences in experimental context from those of the perturbation itself.

This fundamental challenge of extracting meaningful biological insights from perturbation data has spurred the development of diverse computational approaches^10–13^. Most existing approaches focus specifically on predicting the effects of unobserved perturbations^14–21^. This addresses a fundamental limitation of experimental methods: it is physically impossible to perform all possible configurations of perturbation experiments owing to the effectively infinite number of potential experimental designs (considering the time of measurement can be arbitrarily long, the number of experiments that may be conducted is already unbounded based on this dimension alone). For example, the graph-enhanced gene activation and repression simulator (GEARS)^15^ leverages gene representations based on domain knowledge^22^ to predict the effects of unseen genetic perturbations while also providing a means of identifying genetic interaction subtypes. The compositional perturbation autoencoder (CPA)^19^ predicts the effects of unseen perturbation combinations, including drugs as perturbagens and their dosages. Beyond perturbation effect prediction, some methods focus on other critical biological discovery tasks, such as estimating gene–gene relationships^23^, learning transferable cell representations^24,25^, modeling relationships among different types of readout^26–28^ or aiding experimental design^29,30^.

More recently, foundation models^31–34^ have emerged that are pretrained on large collections of transcriptomics data to address multiple biological discovery tasks through task-specific fine-tuning pipelines. These models, exemplified by Geneformer^31^ and scGPT^32^, use Transformer-based encoders^35^ to infer gene and cell representations from gene expression measurements. While their encoder-based approach offers a compelling advantage—the ability to make predictions for previously unseen contexts by extracting contextual information from gene expression profiles—it faces two substantial limitations. First, the low signal-to-noise ratio in high-throughput screens can pose a challenge to the encoder’s ability to extract reliable contextual information, which may result in limited prediction performance. Second, these models are primarily designed for transcriptomics data and are not inherently structured to accommodate diverse perturbation experiments that use other perturbation and readout modalities, such as chemical perturbations or low-dimensional screens measuring cell viability.

To enable in silico biological discovery from a diverse pool of perturbation experiments, we demonstrate that heterogeneous experimental data, regardless of perturbation type or readout modality, can be integrated into a large perturbation model (LPM) by representing perturbation, readout and context as disentangled dimensions. Similar to foundation models^31,32^, LPM is designed to support multiple biological discovery tasks, including perturbation effect prediction, molecular mechanism identification and gene interaction modeling. LPM is trained to predict outcomes of in-vocabulary combinations of perturbations, contexts and readouts. LPM introduces two architectural innovations that support its primary goal of handling heterogeneity in perturbation data. First, LPM disentangles the dimensions of perturbation (*P*), readout (*R*) and context (*C*), representing each dimension as a separate conditioning variable. Second, LPM adopts a decoder-only architecture, meaning it does not explicitly encode observations or covariates. The PRC-disentangled, encoder-free LPM architecture introduces key advantages:

- Seamless integration of diverse perturbation data. By representing perturbation experiments as *P*–*R*–*C* dimensions, LPM effectively learns from heterogeneous experiment data across diverse readouts (for example, transcriptomics and viability), perturbations (CRISPR and chemical) and experimental contexts (single-cell and bulk) without loss of generality and regardless of dataset shape or format.
- Contextual representation without encoder constraints. Encoder-based models assume that all relevant contextual information can be extracted from observations and covariates, which may be limiting due to high variability in measurement scales across contexts and a potentially low signal-to-noise ratio. By contrast, LPM learns perturbation-response rules disentangled from the specifics of the context in which the readouts were observed. A limitation of this approach is the inability to predict perturbation effects for out-of-vocabulary contexts.
- Enhanced predictive accuracy across experimental settings. By leveraging its PRC-disentangled architecture and decoder-only design, LPM consistently achieves state-of-the-art predictive accuracy across experimental conditions.

When trained on a pool of experiments, we demonstrate experimentally that LPM achieves state-of-the-art performance in post-perturbation outcome prediction. In addition, LPM provides meaningful insights into the molecular mechanisms underlying perturbations, readouts and contexts. LPM enables the study of drug–target interactions for chemical and genetic perturbations in a unified latent space, accurately associates genetic perturbations with functional mechanisms and facilitates the inference of causal gene-to-gene interaction networks. To demonstrate the potential of LPM for therapeutic discovery, we used a trained LPM to identify potential therapeutics for autosomal dominant polycystic kidney disease (ADPKD). Finally, we show that the superior performance of LPM compared with existing methods is driven by its ability to leverage perturbation data at scale, achieving significantly improved performance as more data become available for training.

## Results

**Fig. 1:**
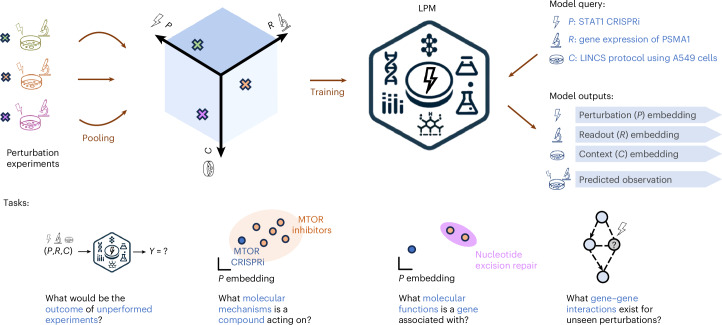
Addressing biological discovery tasks with LPM. Top left: perturbation experiments originating from different studies (green, orange and purple indicate separate experiments) are pooled together. Each experiment is placed in the space spanned by perturbations (*P*), readouts (*R*) and experimental contexts (*C*), where multiple experiments generally only partially overlap in the three-dimensional (*P*,*R*,*C*) space. Central icon: a LPM is trained on pooled perturbation data and can be queried with the symbolic representation of perturbation, readout and context of experiments of interest to generate embeddings and predict outcomes even for configurations that were not observed during training. Top right: trained LPM can be queried to predict experiment outcome given symbolic representations of *P*, *R* and *C* (blue). Bottom: LPM embeddings and predictions carry rich information for a range of biological discovery tasks using transfer learning.

LPM is a deep-learning model that integrates information from pooled perturbation experiments (Fig. 1). We train LPM to predict the outcome of a perturbation experiment based on the symbolic representation of the perturbation, readout and context (the *P*,*R*,*C* tuple). LPM features a PRC-conditioned architecture that enables learning from heterogeneous perturbation experiments that do not necessarily fully overlap in the perturbation, readout or context dimensions. By explicitly conditioning on the representation of an experimental context, LPM learns perturbation-response rules disentangled from the specifics of the context in which the readouts were observed. LPM predicts unseen perturbation outcomes, and its information-rich generalizable embeddings are directly applicable to various other biological discovery tasks (Fig. 1).

### Predicting outcomes of unobserved perturbation experiments

**Fig. 2:**
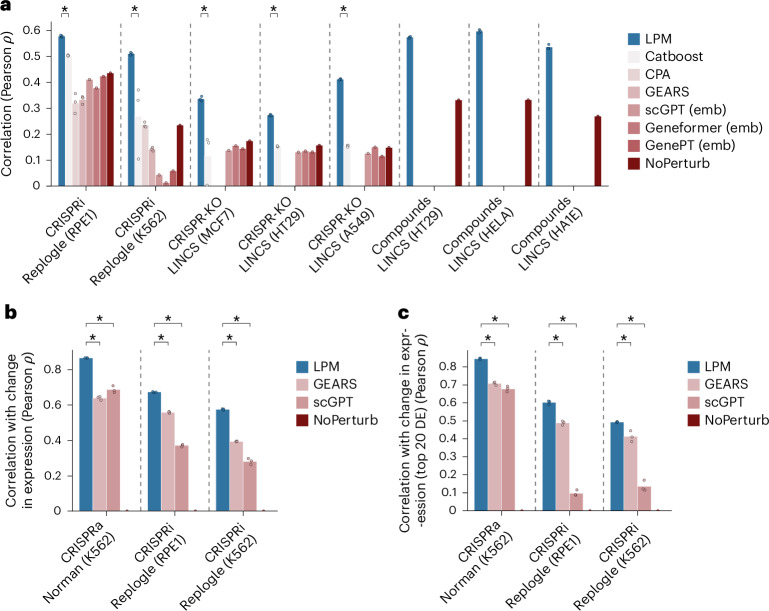
Performance in predicting post-perturbation gene expression. The performance of LPM was compared against state-of-the-art baselines across a variety of experimental settings, contexts and for different perturbation types. **a**, A comparison of methods for post-perturbation expression prediction using *z*-normalized data including all readouts comparing Pearson correlation (*y* axis) on held-out test data from eight experimental contexts (*x* axis) including single-cell (Replogle et al.^9^), bulk (LINCS^7^), genetic (CRISPRi and CRISPR-KO) and chemical compound interventions. **b**,**c**, In addition, we performed a comparison methods for post-perturbation expression prediction that replicates the preprocessing methodology from Roohani et al.^15^ and Cui et al.^32^. In this comparison, we calculated the Pearson correlation between true and predicted changes in log-normalized expression (control versus perturbed) measured on held-out test data for all genes (**b**) and on the subset of the top 20 differentially expressed transcripts (**c**) (*y* axis). Norman et al.^76^ include both single and multiperturbation data. Embedding (‘emb’ in parentheses) next to a baseline indicates that we used embeddings that were fine-tuned using Catboost. For baselines without this indication, we used author instructions for generating the post-perturbation expression predictions. Not all methods are suitable for all settings that LPM operates on and are therefore not included in all comparisons. Asterisks indicate statistical significance (one-sided Mann–Whitney, *P ≤ 0.05). Dots on top of bars represent random seeds. Source data

We evaluated the performance of LPM in predicting gene expression for unseen perturbations against state-of-the-art baselines, including CPA^19^ and GEARS^15^ (Fig. 2). We also included baseline models that combined a Catboost regressor^36^ with existing gene embeddings derived from biological databases (STRING^37^, Reactome^38^ and Gene2Vec^39^), single-cell foundation models based on pooled gene expression data not under perturbations (Geneformer^31^ and scGPT^32^) and natural language descriptions of genes processed through ChatGPT (GenePT^34^). For scGPT and Geneformer, we either fine-tuned the models according to their respective instructions or used their embeddings with a CatBoost model (indicated as ‘emb’). In addition, we included the ‘NoPerturb’ baseline^15^ that assumes that the perturbation does not induce a change in expression. Note that no other baseline model supports predicting outcomes of chemical perturbations and that GEARS, CPA and scGPT (following author instructions) require single-cell-resolved data.

To robustly evaluate the performance of LPM, we conducted a representative array of experiments that covers (1) a range of experimental contexts, (2) different perturbation types (chemical and genetic) and (3) varying preprocessing strategies. Across all studied experimental settings, LPM consistently and significantly outperformed the state-of-the-art baselines, regardless of preprocessing methodology. Further data from Horlbeck et al.^40^, which included viability readouts for pairwise CRISPRi perturbations, are presented in the Supplementary Information to demonstrate that LPM is effective even in low-dimensional settings with nontranscriptomic readouts. For details on the datasets and their preprocessing, see the Methods.

### Mapping a compound-CRISPR shared perturbation space

**Fig. 3:**
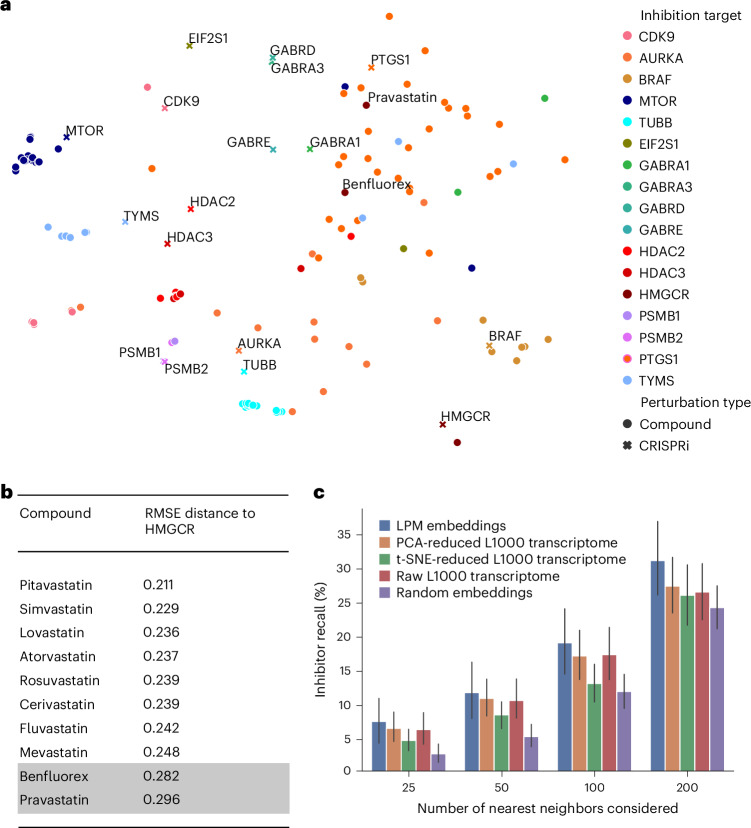
Learning compound-CRISPR perturbation representations. **a**, The latent space of compound and CRISPR knockouts (reduced to two-dimensions via t-SNE) reflects known groupings of compound and genetic perturbations that target the same molecular mechanisms in bulk LINCS L1000 data from ref. ^7^. Genes targeted by corresponding CRISPR and compound inhibitors are color-coded in matching colors. **b**, Root mean squared error (RMSE) distances of known *HMGCR* inhibitors (statins) to the corresponding CRISPR-*HMGCR* perturbation in the embedding space of the LPM. Two bottom outliers are additionally annotated in **a**: benfluorex (withdrawn for cardiovascular side effects^42^) and pravastatin (shown to have low correlation to other statins^78^ and additional anti-inflammatory effects^44–46^). **c**, The RMSE-based distance between perturbation embeddings for CRISPR perturbations was used to measure the recall of known inhibitors of the respective genetic target, for different numbers of nearest neighbors. We compared LPM embeddings with those derived from post-perturbation L1000 transcriptome profiles. Bars represent the 95% confidence intervals across genetic targets (*N* = 89). Source data

To evaluate the ability of LPM to support the generation of insights across different types of perturbation, we trained an instance of LPM using all available data from Library of Integrated Network-Based Cellular Signatures (LINCS) experiments^7^ involving both genetic and pharmacological perturbations across a total of 25 experimental contexts with unique combinations of cellular contexts and perturbation types. LPM integrates genetic and pharmacological perturbations within the same latent space, enabling the study of drug–target interactions. When studying *t*-distributed stochastic neighbor embeddings (t-SNE)^41^ of the perturbation embedding space learned by the LPM, we found that pharmacological inhibitors of molecular targets are consistently clustered in close proximity to genetic CRISPR interventions that target the same genes (Fig. 3a). For example, genetic perturbations targeting *MTOR* and compounds inhibiting *MTOR* and also genetic perturbations targeting genes from the same pathway, for example *PSMB1* and *PSMB2*, or *HDAC2* and *HDAC3*, were clustered closely together. Qualitatively, we found that anomalous compounds that were placed distant from their putative target had been reported to have off-target activity (Fig. 3b), such as benfluorex (withdrawn due to cardiovascular side effects^42^) and pravastatin (shown to elicit expression changes with low correlation to other statins^43^). Intriguingly, we found that pravastatin moved toward nonsteroidal anti-inflammatory drugs that target gene *PTGS1* in the perturbation space (Fig. 3a), indicating a potential additional anti-inflammatory mechanism of pravastatin. We found that this movement independently derived by LPM is indeed substantiated by clinical and preclinical observations that ascribe anti-inflammatory effects to pravastatin^44–46^. To further quantitatively validate these findings, we systematically compared known inhibitors of a genetic target with the genetic perturbation in embedding space as a reference. We evaluated the neighborhood of the reference in various embedding spaces and found that perturbation embeddings derived from LPM achieve considerably higher recall of known inhibitors of genetic targets compared with embeddings derived from post-perturbation L1000 transcriptome profiles or dimensionality reduced versions thereof (Fig. 3c).

### Learned embeddings reflect known biological relationships

**Fig. 4:**
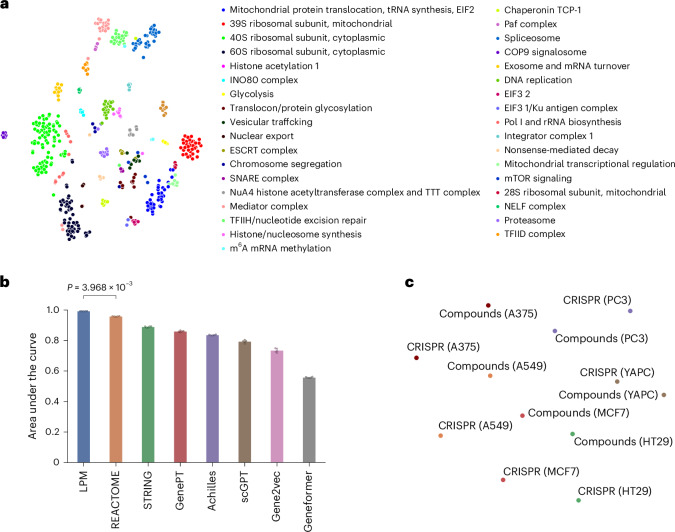
Biological relationships captured in LPM embeddings. **a**, LPM perturbation (*P*) embeddings (t-SNE embedded in two-dimensional (2D) space). Each point represents a CRISPRi perturbation color-coded by the molecular function of its respective genetic target from ref. ^9^. **b**, Performance of LPM perturbation (*P*) embeddings compared with existing state-of-the-art gene embeddings derived from large-scale genetic screens and public pathway and interaction databases in predicting gene function annotations from ref. ^9^ (*P* value calculated via one-sided Mann–Whitney test). Dots on the top of bars represent replicates across *N* = 5 random seeds. **c**, LPM context (*C*) embeddings (2D t-SNE representation) quantify similarity between experimental contexts. Intriguingly, we found that contexts are grouped with respect to the model system under study (shown in the figure) or by type of perturbation (not shown), depending on the t-SNE random seed used. Source data

To evaluate the degree to which LPM perturbation embeddings correspond to known biological functions, we extracted perturbation embeddings for well-characterized perturbations from an LPM trained on pooled single-cell perturbation data^9^ and compared genetic perturbations with gene function annotations as curated by Replogle et al.^9^ using the comprehensive resource of mammalian protein complexes (CORUM)^47^ and search tool for recurring instances of neighbouring genes (STRING)^37^ databases. We found that LPM implicitly organizes perturbations according to their molecular functions (Fig. 4a) and that these embeddings are significantly (*P* ≤ 0.01) more predictive of gene function annotations than existing state-of-the-art gene perturbation embeddings (Fig. 4b), including those derived from curated databases such as STRING^37^ and Reactome^38^, derived from co-expression datasets in Gene2Vec^39^ and derived from the single-cell unperturbed gene expression foundation models Geneformer^31^ and scGPT^32^ and gene embeddings based on natural language descriptions processed through ChatGPT (GenePT^34^).

To qualitatively assess the information contained within context representations of LPM, we used the LPM model trained on combined LINCS data from the perturbation embedding experiment above to generate context embeddings. We found that—depending on the t-SNE random seeds used—either cell types tend to cluster together with matching cell types from other experiments (Fig. 4c), or the context embeddings tend to cluster based on the perturbation methodology (CRISPR versus compound screens; not depicted). The qualitative results imply that the information contained within the learned context embeddings carries information regarding biological semantics and could thus be valuable in downstream analyses, such as for quantifying the similarity of contexts.

### In silico discovery of candidate therapies for ADPKD

**Fig. 5:**
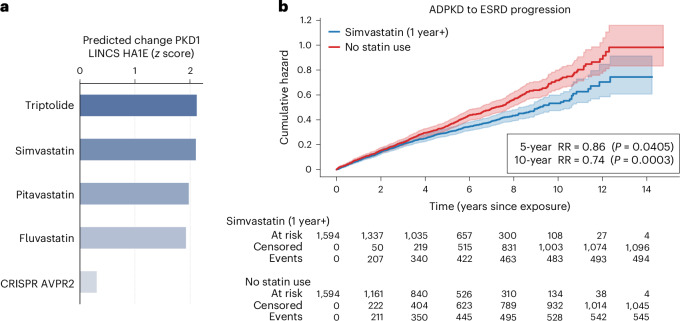
In silico discovery of potential therapeutics for ADPKD. **a**, Using LPM, we conducted an in silico perturbation study in which we identified clinical-stage drugs that are predicted to upregulate *PKD1* in embryonic kidney cells. A lack of functional copies of *PKD1* is hypothesized to be causally involved in ADPKD pathogenesis and progression^49–51^. We found that triptolide, simvastatin (bold) and other statins, are the top predicted upregulators of *PKD1* among clinical-stage drugs. For reference, we also include the predicted CRISPRi on vasopressin receptor 2 (*AVPR2*) to simulate the effect of the FDA-approved AVPR2 antagonist tolvaptan^79,80^ that is mechanistically distinct^81,82^. Bars represent model predictions in the form of *z* scores. **b**, Because simvastatin is commonly prescribed for cardiovascular indications, we were able to conduct a retrospective cohort study in large-scale electronic health records to further substantiate the potential efficacy of simvastatin in reducing ADPKD progression in the clinic. Most notably, we found that—among individuals diagnosed with ADPKD—1 year or longer exposure to simvastatin (blue) is associated with a significant (*P* ≤ 0.05, 5-year relative risk (RR) of 0.86 and 10-year RR of 0.74) reduction in progression to ESRD compared with those not exposed to statins (red). The 95% confidence intervals were estimated using the Nelson–Aalen estimator^83,84^.

We hypothesized that the ability of LPM to conduct perturbation experiments in silico with high accuracy while reflecting underlying biological function could be used to discover potential candidate therapeutics for diseases with known genetic causes, such as ADPKD. ADPKD is a genetic disease suspected to be caused by mutations in *PKD1*^48^ that are reported to lead to a lack of functional *PKD1*—eventually manifesting in dose-dependent cystogenesis^49–52^. ADPKD affects more than 12 million people worldwide^53^ and may lead to severe long-term complications, such as end-stage renal disease (ESRD) and the dependence on dialysis or a kidney transplant. There are no curative treatments available for ADPKD. A potential hypothesis for a therapeutic could be to upregulate expression of the functional allele of *PKD1* in heterozygous carriers of *PKD1* mutations to make up for the nonfunctional allele and thereby reach a sufficient level of functional *PKD1* that may inhibit further progression of ADPKD. To identify potential therapeutics that could increase *PKD1* expression in individuals with ADPKD, we conducted an in silico perturbation experiment using an LPM trained on pooled LINCS compound and genetic perturbation data to predict which clinical-stage drugs may lead to upregulation in *PKD1* levels in HA1E embryonic kidney cells cultured under the LINCS L1000 protocol^54^. We found that triptolide, simvastatin and other statins were among the top clinical-stage drugs predicted to cause increased *PKD1* expression in vitro (Fig. 5a). Our findings align well with previous literature, where effects of commercially available statins were shown to increase the expression of *PKD1* in pancreatic cancer cell line MiaPaCa-2^55^. We note that Huang et al.^56^ found no significant change in *PKD1* expression in mice exposed to atorvastatin. As simvastatin is a Food and Drug Administration (FDA)-approved medicine that is prescribed preventatively for cardiovascular indications, we conducted a retrospective, matched cohort study^57,58^ using a non-linear propensity score estimator^59^ to validate the in silico hypothesis that simvastatin may lead to reduction in ESRD progression in real-world clinical data from the Optum deidentified Electronic Health Record database. Notably, we found that—among individuals diagnosed with ADPKD^60^—exposure to simvastatin over 1 year or longer was associated with a significant decrease (5-year relative risk 0.86, *P* = 0.0405, and 10-year relative risk 0.74, *P* = 0.0003) in progression to ESRD^61^ compared with those not exposed to any statins predicted by LPM to increase expression of *PKD1* (Fig. 5b). Several of the therapeutics predicted to increase *PKD1* are substantiated by literature; for example, pravastatin was shown to be associated with improved kidney markers in a clinical study in young individuals^62^, and triptolide led to a reduction of cystogenesis in murine models^63,64^. *PKD1* was neither measured nor perturbed in LINCS, the 5,310 chemical perturbations were not all tested in HA1E cells, and the in silico LPM experiments were therefore essential to enable this study. We note that these findings should not be considered definitive and that further research is required to validate and support them.

### Facilitating inference of causal gene–gene relationships

**Fig. 6:**
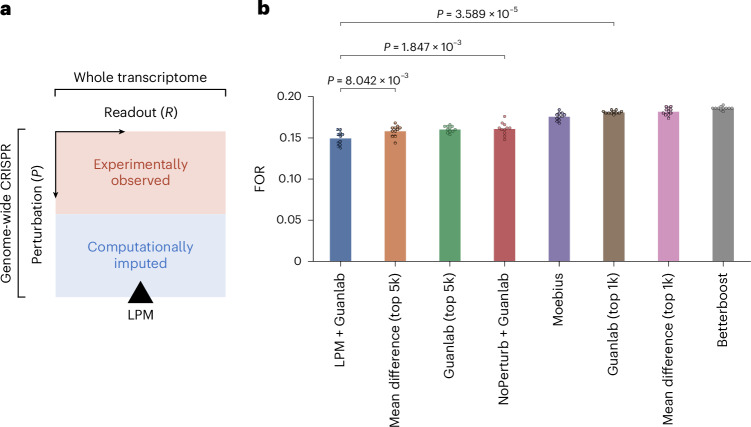
Improved gene–gene network inference with LPM. **a**, We used LPM to predict post-perturbation transcriptomes for unseen perturbations, completing a partially observed experimental space where only half (orange area) of the possible CRISPRi perturbations across the genome were experimentally observed. We hypothesized that access to the computationally completed dataset (blue plus orange area) may enable the state-of-the-art Guanlab^85^ gene network inference method to more accurately infer the gene–gene interactions for genes not experimentally perturbed. **b**, We found that the combination of LPM imputation and the Guanlab method (LPM+Guanlab) significantly outperformed existing methods for gene–gene network inference using the partially observed dataset alone in terms of FOR in a gene network inference benchmark^23^ using single-cell data from Replogle et al.^9^. Dots on top of bars represent replicates across *N* = 11 random seeds (*P* values calculated using one-sided Mann–Whitney–Wilcoxon). Source data

To assess to what degree the accuracy of the predictions of LPM translate to capturing mechanistic interactions between genes, we used LPM in the context of causal inference of gene interaction networks. Normally, these networks are inferred from perturbation experiments in which only a subset of all genes were perturbed. By contrast, we measured the enhancement in performance when those networks were inferred from the same experimental data enriched with missing, unmeasured CRISPRi perturbations predicted in silico using LPM. In particular, to perform network inference, we applied corresponding methods that demonstrated best-in-class performance on the recent CausalBench challenge^23,65^ and were designed specifically for inferring gene–gene networks from perturbational single-cell RNA sequencing data. We found that augmenting the original data with in silico perturbation outcomes, before applying network inference using above-mentioned methods, leads to a significant improvement in terms of false omission rate (FOR) in comparison with existing state-of-the-art methods for gene–gene network inference that do not have access to perturbation imputation (Fig. 6). These results underscore the utility of LPM in supporting the inference of more comprehensive and accurate causal interactions tailored to a given experimental context and the ability of LPM to learn generalizable, causal interactions between perturbations.

### LPM performance improves with more training data

**Extended Data Fig. 1:**
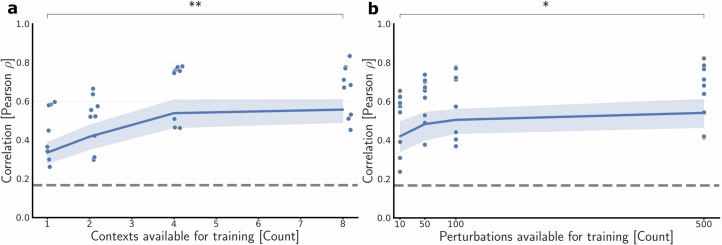
Performance of LPM as a function of training data availability. Performance comparison in terms of Pearson correlation coefficient *ρ* in predicting the outcomes of unseen experiments of LPM when varying **a**. the number of perturbations available for training in a target context and **b**. the number of different contexts available for training. Dots correspond to individual runs with a different random seed, and the blue line corresponds to the inferred trend (the average value with SD depicted in shaded blue). The dashed grey line denotes the performance of the ‘NoPerturb’ baseline, which does not take perturbation information into account. The performance of LPM is significantly increased (* = p ≤0.05, ** = p ≤0.01; one-sided Mann-Whitney test) when more perturbations and more contexts are available.

In contrast to data-rich domains such as natural language processing, where scaling of model performance with additional data has been studied experimentally^66,67^, it is not yet clear to what degree in silico biological discovery can benefit from the availability of additional data across both contexts and perturbations for pooling. Establishing data scaling patterns in biology has historically been more difficult than in predominantly digital domains such as natural language processing and computer vision because biological perturbation data can often not be naively aggregated owing to the intricate connection between experimental context, data processing methodologies and batch effects^68,69^. To elucidate the potential performance benefits of additional data for LPM, we computationally evaluated the prediction performance in terms of Pearson correlation coefficient *ρ* for predicting unseen perturbations when varying the number of datasets covering multiple contexts and perturbations in a single context available for model training (Extended Data Fig. 1). The performance of LPM significantly (*P* ≤ 0.05) improves both when more datasets covering multiple contexts and when more perturbations in a single context are available for training.

## Discussion

LPM demonstrates that integrative learning across heterogeneous perturbation screens can deliver accurate, in silico estimates of perturbation-, readout- and context-specific experimental outcomes. We found that the use of LPM—either independently or in combination with a causal network inference algorithm—significantly outperforms existing state-of-the-art methods, providing an experimental proof of concept for the potential to accelerate biological discovery with computationally generated evidence. The ability to generate unobserved experimental data for critical biological questions, such as what the estimated effects of unseen perturbations would be, could accelerate the generation of insights and complement experimentally generated data—particularly in settings that are difficult, time-intensive or resource-intensive to study in real-world laboratory experiments. Notably, we found that LPM implicitly learns rich latent space embeddings for perturbations, readouts and experimental contexts as is required to achieve their explicit training objective to predict yet unseen experimental outcomes. The rich latent space embeddings of LPM enables a range of downstream biological discovery tasks (only a subset of the potential use cases are investigated in this study), which demonstrates the versatility and multitask capability of LPM that captures underlying mechanistic relationships in data.

LPM still faces important limitations. First, the training data used in our study are publicly available and sufficiently standardized; however, non-immortalized cell lines, rare cell types, primary tissues and patient-derived samples remain underrepresented. Second, the model can interpolate and handle symbols within its training vocabulary but cannot yet extrapolate to unseen symbols—for instance, novel cell types or perturbations—unless suitable pretrained embeddings are explicitly supplied. Nevertheless, recent trends indicate that in the near future, as perturbational experimental data becomes more abundant, the experimental space will be sufficiently covered, rendering in-vocabulary approaches sufficient for most tasks. Third, hidden batch effects, inconsistent preprocessing and incomplete metadata can still erode performance, as in other large-scale biological models. Fourth, the ADPKD case study is retrospective and therefore vulnerable to unobserved confounders; mechanistic conclusions will remain provisional until prospective validation. As a further limitation, we considered only a single genetically validated marker in our ADPKD study but therapeutic candidates must be optimized with regard to multiple criteria, including safety, pharmacokinetics and pharmacodynamics. It is important to note that further clinical validation is needed to conclusively establish causality for the predictions of LPM in the context of ADPKD. Finally, we would like to emphasize that gene network inference is a distinct and complex field of research^70^, and future studies will need to explore additional datasets and benchmarks to further validate findings in this area. Our study, however, is focused on demonstrating the potency of high-quality perturbation-effect predictors, such as LPM, to complement existing network inference methods.

Several experimental directions could address these gaps, including prospective perturbation screens in primary and patient-derived cells to test whether LPM maintains accuracy outside immortalized lines and under different dosages^71^. Applying LPM to data derived from clinical settings could present valuable opportunities to identify novel therapies or patient cohorts that are likely to respond to specific treatments, thus advancing the field of personalized medicine. By leveraging these datasets, LPM could help to pinpoint biomarkers of response (for example, clinical covariates) and further optimize therapeutic strategies for patients based on their unique molecular profiles. In general, if curated and standardized perturbation data continue to grow, parameter-scaling results suggests that larger LPM variants could yield proportional gains in predictive accuracy and mechanistic resolution. Systematic efforts to reduce batch effects and harmonize metadata will be as important as algorithmic advances in realizing that potential.

## Methods

### Problem formulation

We consider every experimental system subject to a perturbation (represented symbolically) for which we observe a readout. For example, an experiment could be conducted in a single-cell in vitro system in which transcript counts are measured after CRISPRi targeting a specific gene. A biological model system is considered to be a black box, and no prior knowledge is assumed about the internal mechanism that gives rise to observed readouts.

The totality of the experimental context, including model system under study and the experimental protocol used, is represented by the variable $C\in {\mathcal{C}}$ and is referred to as the context of the experiment. The context *C* is a symbolic description of the system itself and implicitly represents all the covariates that constitute the experimental conditions, for example, biological context details such as cell type, genetic background and incubation protocols. We consider a perturbation to be any input to the system that is not already included in the context, including a chemical compound, a gene knockout or a disease that has perturbed the system are examples of perturbations. Let $P\in {\mathcal{P}}$ be the vector that describes a perturbation. Similar to the context *C*, *P* is a symbolic representation of the perturbation. For instance, CRISPRi_STAT1 would symbolically represent CRISPR interference of gene *STAT1*. In addition, multiperturbations that are symbolically represented as, for example, CRISPRi_STAT1+CRISPRa_FOXF1 (CRISPR interference of gene *STAT1* coupled with CRISPR-mediated transcriptional activation of *FOXF1*), are modeled as a function of corresponding embeddings. In the experiments in this Article, we used the embedding average. The symbolic description of the measurements observed in the system that is under perturbation is represented by a readout $R\in {\mathcal{R}}$, where ${\mathcal{R}}$ is a set of symbols that correspond to all possible discrete values that represent observed readouts. For example, *R* can represent the gene expression of the gene *PSMA1*, denoted as Transcript_PSMA1. The concrete measurement taken in context *C* after perturbation *P* using readout *R* is represented by $Y\in {\mathcal{Y}}\subseteq {\mathbb{R}}$. It is notable that the experimental observation *Y* is distinct from the readout *R* in that *R* symbolically describes the type of measurement taken, whereas *Y* is a concrete instance of that measurement in the experimental context *C* under perturbation *P*.

Let *O* = (*P*, *R*, *C*, *Y*) be the stack of aforementioned random variables and ${\mathcal{I}}=\{1,2,\ldots \}$ be the index set of all possible potential observed samples. Therefore, the index $i\in {\mathcal{I}}$ refers to one potential observation *O*^(*i*)^ = (*P*^(*i*)^, *R*^(*i*)^, *C*^(*i*)^, *Y*^(*i*)^). Let ${{\mathcal{D}}}_{{\rm{obs}}}=\{{O}^{(1)},{O}^{(2)},\ldots ,{O}^{({n}_{{\rm{obs}}})}\}$ be the set of observations that has *n*_obs_ data points and ${{\mathcal{I}}}_{{\rm{obs}}}\subseteq {\mathcal{I}}$ be the set of associated indices. It is clear that *Y* is not independent from *P*, *R* and *C*. We want to learn the causal model *q*(*Y*∣*do*(*P* = *p*), *R*, *C*). Here, *q* is the probability distribution of the outcome *Y* in a biological system within the context *C* when the perturbation *p* is applied and the readout *R* is observed. We would like to leverage the structural dependence between these variables to estimate *q* from ${{\mathcal{I}}}_{{\rm{obs}}}$ so it can predict the outcome of unobserved (perturbation, readout and context) combinations indexed by $j\in {{\mathcal{I}}}_{{\rm{unobs}}}={\mathcal{I}}\backslash {{\mathcal{I}}}_{{\rm{obs}}}$. Mathematically, we want to estimate

1 $$q(Y| P,R,C,{{\mathcal{I}}}_{{\rm{obs}}})$$

for any combination $(P,R,C)\in {\mathcal{P}}\times {\mathcal{R}}\times {\mathcal{C}}$. This is possible only if the spaces ${\mathcal{P}}$, ${\mathcal{R}}$ and ${\mathcal{C}}$ have some structure that allows the concept of distance to be defined. For example, for a system with context *C*^(*j*)^, predicting the effect of perturbation *P*^(*j*)^ on readout *R*^(*j*)^ is possible if the outcome of a similar perturbation on a similar readout is already observed for a system within a similar context. Clearly, discussing similarities requires the relevant spaces to possess some structure in which a distance metric can be defined. As (*P*, *R*, *C*) are in essence discrete symbolic values, it is necessary to first transform them into more tractable spaces that we call embedding spaces. Let ${Z}_{P}\in {{\mathcal{Z}}}_{P}\subseteq {{\mathbb{R}}}^{{d}_{{Z}_{P}}}$, ${Z}_{R}\in {{\mathcal{Z}}}_{R}\subseteq {{\mathbb{R}}}^{{d}_{{Z}_{R}}}$ and ${Z}_{C}\in {{\mathcal{Z}}}_{C}\subseteq {{\mathbb{R}}}^{{d}_{{Z}_{C}}}$ be the random variables that represent the embeddings of *P*, *R* and *C*, respectively. The transformation maps ${\phi }_{p}:{\mathcal{P}}\to {{\mathcal{Z}}}_{P}$ ${\phi }_{r}:{\mathcal{R}}\to {{\mathcal{Z}}}_{R}$ and ${\phi }_{c}:{\mathcal{C}}\to {{\mathcal{Z}}}_{C}$ that induce such structure in the embedding spaces are learned from ${{\mathcal{I}}}_{{\rm{obs}}}$. In other words, the information of the observed data is learned in *ϕ*_*p*_(⋅), *ϕ*_*r*_(⋅) and *ϕ*_*c*_(⋅) functions. This means that, for any unseen (*P*, *R*, *C*) tuples, their corresponding embeddings *Z*_*P*_, *Z*_*R*_ and *Z*_*C*_ implicitly contain some information from ${{\mathcal{I}}}_{{\rm{obs}}}$. This is indeed the reason that enables knowledge transfer to unseen perturbations, readouts and contexts. With the learned embedding space, equation (1) can be written as

2 $${q}_{{\rm{emb}}}(Y| {Z}_{P},{Z}_{R},{Z}_{C},{{\mathcal{I}}}_{{\rm{obs}}}),$$

where the subscript ‘emb’ emphasizes that the map is defined from the embedding spaces instead of the original spaces. Due to the learned structure in the embedding spaces, it is expected that *q*_emb_(⋅) be more accessible to learn than *q*(⋅).

### Model architecture

**Extended Data Fig. 2:**
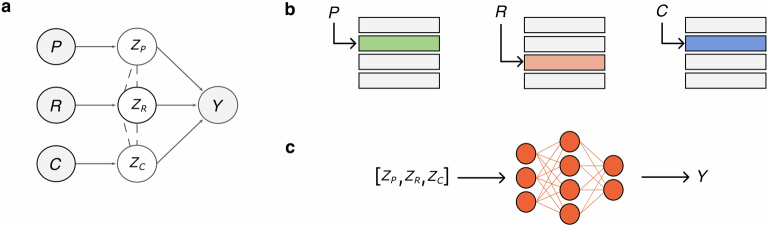
Model architecture. **a**. Graphical model shows the dependencies between random variables previously described in Section 4.1. Dashed lines indicate implicit bi-directional dependencies that enable transfer learning across datasets. Symbolic perturbation, readout, and context descriptors (*P*,*R*,*C*) are first embedded (*Z*_*P*_, *Z*_*R*_, *Z*_*C*_), then used to generate output *Y* that represents the value of the readout *R*. **b**. Embeddings are implemented as learnable look-up tables. *P*, *R*, and *C* identify indices in the corresponding tables. **c**. Concatenated embeddings are forward propagated through a multilayer perceptron to predict the output *Y*

Building on the problem formulation described in the ‘Problem formulation’ section, we designed the architecture of LPM as shown in Extended Data Fig. 2a. Because *P*, *R* and *C* are discrete random variables, it is simple to implement the corresponding embeddings *Z*_*P*_, *Z*_*R*_ and *Z*_*C*_ using symbol vocabularies and learnable look-up tables (Extended Data Fig. 2b). Symbol vocabularies map symbols to indices, while look-up tables map indices to learnable weights that we treat as embeddings. This model can nevertheless be trivially generalized to include more complex perturbations or context descriptions; for example, multiple perturbations can be implemented as a sum of individual perturbations^15,19^. The prediction network is a neural network that is learned end-to-end together with the embeddings, by backpropagating the error using the Adam optimizer^72^. We found the multilayer perceptron architecture with ReLU activation functions, implemented on top of concatenated embeddings, to work satisfactorily (Extended Data Fig. 1c). We note that an extensive architecture search was not performed and further architecture tuning could potentially further improve results.

The key property of our model that enables scaling training across heterogeneous high-throughput perturbation screens (Fig. 1) is its conditioning on the readout *R*. To clarify why this simple trick is effective, consider an alternative description of the causal model equation (1) that does not condition on the *R*, that is, $q{\prime} (Y| P,C,{{\mathcal{I}}}_{{\rm{obs}}})$ (ref. ^73^). In this case, $Y\in {\mathcal{Y}}\subseteq {{\mathbb{R}}}^{{d}_{y}}$ is a vector (not a scalar) whose dimension *d*_*y*_ is the number of readouts. The challenge is that de facto each perturbation screen has its own subset of phenotypic readouts. Even when the same modality—such as the transcriptome—is measured in two datasets, they often capture different subsets of that modality. The problem exacerbates if different modalities are used for training (for instance, proteome along with transcriptome), or if a large number of datasets is included in the training process. Related previous works alleviate this issue by selecting only readouts that appear in all considered perturbation experiments. However, this approach is clearly suboptimal because it discards relevant information. Moreover, it becomes impractical when scaling to many datasets, because the size of the overlapping feature set shrinks as the number of datasets increases. Moreover, certain experimental measurement technologies, such as DRUG-seq^74^, may contain missing values. LPM is designed to be robust to missing readouts as well.

### Data sources

The datasets used for benchmarking include single-cell and bulk data, genetic (CRISPRi, CRISPR activation (CRISPRa) and CRISPR-knockout (CRISPR-KO)) and chemical compound perturbations, and single- and multiperturbation settings. The full overview of all used data is presented in the Supplementary Information. Single-perturbation single-cell data contain two experimental contexts from ref. ^9^ : Replogle et al. (K562) and Replogle et al. (RPE1). The data are based on transcriptome measurements generated after DepMap essential genes^75^ have been perturbed using CRISPRi Perturb-seq technology. The data were sequenced in chronic myeloid leukemia (K562) and retinal pigment epithelial (RPE1) cell lines, respectively. In the single-cell space, we also used multiperturbation experiments of type CRISPRa from Norman et al.^76^, which were performed also on K562 cells using Perturb-seq. For bulk data, we used the expanded Connectivity Map Lincs 2020 screens (https://clue.io)^7^, on both pharmacological and CRISPR-KO perturbations. A total of 26 biological contexts from LINCS studies based on bulk data were used, encompassing different cell and perturbation types. We discarded LINCS contexts that had too few perturbations (<300), for simplicity, as they did not make a difference in our analysis. To further simplify the analysis, we used only the most commonly appearing drug doses (10 μM) and observation times (24 h).

### Data preprocessing

We used two preprocessing approaches to test robustness and perform a fair comparison against competing methods. In our first set of experiments, for single-cell data from ref. ^9^, we used the *z*-normalized version of the datasets, as recommended and provided by the authors. For single-perturbation single-cell data, *z* normalization was performed per gemgroup (batch). Single-guide RNAs that target the same gene were aggregated to represent a single perturbation. We removed cells containing multiple knockdowns to simplify the evaluation, focusing exclusively on predicting unobserved perturbations rather than combinations of observed perturbations. For bulk data, we used the preprocessed data that included quality control as provided by Subramanian et al.^7^ (level 5, phase II data). We kept only 978 experimentally measured readouts and dropped inferred gene expressions. In our second set of experiments, we used data from both single-perturbation experiments Replogle et al. (K562) and Replogle et al. (RPE1), as well as multiperturbation experiments from Norman et al.^76^ processed as described in ref. ^15^ (log-transformed and filtered to 5,000 highly variable genes). This preprocessing strategy is arguably the most established in the literature for evaluating perturbation models.

### Benchmarking

As a part of our benchmarking, we compared LPM against six baselines: (1) CPA^19^, (2) GEARS^15^, (3) CatBoost^36^ combined with precomputed gene embeddings from STRING^37^, Reactome^38^ and Gene2Vec^39^, (4) Geneformer^31^, (5) scGPT^32^ and (6) GenePT^34^. Geneformer and scGPT were either fine-tuned according to the authors’ instructions or used as frozen embedding generators (suffix ‘emb’). The NoPerturb baseline^15^ was included as a perturbation-agnostic control. For performance benchmarks (Fig. 2), we used cross-validation and held out a single experimental context as the target context for each fold. Within the target context, test and validation data were randomly held out (stratified by perturbation) and excluded from training, while the remaining target context data and all data from nontarget contexts were used to train LPM (experimental details provided in the Supplementary Information). For GEARS and Catboost-based models, only data from the target context were used because including additional contexts did not benefit those methods. For CPA, due to architectural constraints, we could only include single-cell data from the same experimental studies (that is, all Replogle data). For each target context, we trained models for different random seeds to quantify uncertainty. The remaining details of our experiments are given in the Supplementary Information. They include hyperparameter selection, learning details, baselines and metrics used, and details related to specific downstream tasks.

### Statistics and reproducibility

No statistical method was used to predetermine sample size. The experiments were not randomized. Data collection and analysis were not performed blind to the conditions of the experiments. Source code is available in the code repository^77^.

### Reporting summary

Further information on research design is available in the Nature Portfolio Reporting Summary linked to this article.

## Supplementary information

Supplementary Information Supplementary text, Supplementary Tables 1–4 and Supplementary Figs. 1 and 2.

Reporting Summary

## Source data

Source Data Fig. 2 Source data accompanying the corresponding figure.

Source Data Fig. 3 Source data accompanying the corresponding figure.

Source Data Fig. 4 Source data accompanying the corresponding figure.

Source Data Fig. 6 Source data accompanying the corresponding figure.

## Data Availability

Perturbation data used in this study are from publicly available sources, including Norman et al.^76^ (GSE133344), Replogle et al.^9^ (Figshare), Horlbeck et al.^40^ (GSE116198) and Subramanian et al.^7^ (https://clue.io/). The Optum deidentified Electronic Health Record database used to validate in silico findings in real-world data is available for accredited researchers from Optum, but third-party restrictions apply to the availability of these data. The data were used under license for this study with restrictions that do not allow the data to be redistributed or made publicly available. Data access to the Optum deidentified Electronic Health Record database may require a data sharing agreement and may incur data access fees. Source data are provided with this paper.

## Code availability

Source code is available via GitHub at https://github.com/perturblib/perturblib and via Zenodo at 10.5281/zenodo.15671137 (ref. ^77^).
