# Supplementary material for: In silico biological discovery with large perturbation models
Source: Nat Comput Sci. 2025 Oct 15;5(11):1029–40. doi: 10.1038/s43588-025-00870-1 (PMC12638242; doi:10.1038/s43588-025-00870-1)
Supplement: Supplementary file 1 — Supplementary text, Supplementary Tables 1–4 and Supplementary Figs. 1 and 2. [file 43588_2025_870_MOESM1_ESM.pdf]

nature computational science

Supplementary information https://doi.org/10.1038/s43588-025-00870-1

In silico biological discovery with large perturbation models

In the format provided by the authors and unedited

Supplementary information

Data sources

Biological context (C)	Perturbation type	#Cells (N)	#Readouts (R)	#Perturbations (P)
Replogle et al. (K562) [9]	CRISPRi	310 385	8 562	2 056
Replogle et al. (RPE1) [9]	CRISPRi	247 914	8 748	2 392
Norman et al. (K562) [30]	multi-CRISPRa	110 000	8 241	287
LINCS (A375) [7]	CRISPR-KO	n/a - bulk	978	1 904
LINCS (A549) [7]	CRISPR-KO	n/a - bulk	978	1 917
LINCS (AGS) [7]	CRISPR-KO	n/a - bulk	978	1 464
LINCS (BICR6) [7]	CRISPR-KO	n/a - bulk	978	1 659
LINCS (ES2) [7]	CRISPR-KO	n/a - bulk	978	2 117
LINCS (HT29) [7]	CRISPR-KO	n/a - bulk	978	1 385
LINCS (MCF7) [7]	CRISPR-KO	n/a - bulk	978	1 049
LINCS (PC3) [7]	CRISPR-KO	n/a - bulk	978	772
LINCS (U251MG) [7]	CRISPR-KO	n/a - bulk	978	2 419
LINCS (YAPC) [7]	CRISPR-KO	n/a - bulk	978	994
LINCS (A375) [7]	Compounds	n/a - bulk	978	1 803
LINCS (A549) [7]	Compounds	n/a - bulk	978	1 484
LINCS (ASC) [7]	Compounds	n/a - bulk	978	391
LINCS (HA1E) [7]	Compounds	n/a - bulk	978	1 463
LINCS (HCC515) [7]	Compounds	n/a - bulk	978	1 067
LINCS (HELA) [7]	Compounds	n/a - bulk	978	492
LINCS (HEPG2) [7]	Compounds	n/a - bulk	978	598
LINCS (HT29) [7]	Compounds	n/a - bulk	978	1 082
LINCS (MCF10A) [7]	Compounds	n/a - bulk	978	524
LINCS (MCF7) [7]	Compounds	n/a - bulk	978	2 035
LINCS (MDAMB231) [7]	Compounds	n/a - bulk	978	384
LINCS (NPC) [7]	Compounds	n/a - bulk	978	482
LINCS (PC3) [7]	Compounds	n/a - bulk	978	2 307
LINCS (THP1) [7]	Compounds	n/a - bulk	978	342
LINCS (VCAP) [7]	Compounds	n/a - bulk	978	938
LINCS (YAPC) [7]	Compounds	n/a - bulk	978	414

Supplementary Table 1 **Data overview.** The names in the brackets of experimental contexts indicate cell types. Perturbation counts were computed after the cells that were perturbed with multiple gRNAs targeting different genes were removed. All LINCS contexts have 978 readouts (L1000 transcriptome).

Benchmarking

Perturbation prediction. We evaluated how well LPM performs in terms of predicting expected response to different "unobserved" perturbations. For datasets with single-cell readouts, LPM was trained on mean-aggregated data to reflect effects across cell populations.

We randomly selected 8 biological contexts, covering both bulk and single-cell data, as well as pharmacological and genetic interventions. Perturbation data in each considered biological context (Supplementary Table 1) were split into 70%, 15% and 15% folds for model training, validation and testing, respectively. We performed cross-validation, where in each fold, the test data from one of the considered biological contexts was isolated and the rest of the data (all the data from Supplementary Table 1) was used for training the LPM. For baselines with predefined embeddings, we used only the training data from the target biological context (including other data was not found to affect performance). Validation sets were used for hyper-parameter selection and early stopping.

Model predictions were evaluated against the ground truth with respect to: (i) Root mean squared error (RMSE), (ii) the coefficient of determination (R^2), (iii) Pearson correlation coefficient (Pearson), and (iv) Mean absolute error (MAE). Additionally, we evaluated performance on different portions of test data. We have included the full test set but also 25% and 10% portions of the test data that contained the strongest perturbations and most moving readouts. The magnitude of perturbations and the magnitude of readout movements were measured with respect to RMSE against mean readout values in the control data. The following competing methods were examined:

- **NoPerturb:** This baseline is similar to the one described by [11]. When predicting the outcome of a perturbation, it takes no perturbation information into account but assigns each readout the average value as observed in the training data.

- **Catboost:** We used Catboost algorithm [31] in combination with different predefined embeddings (explained in more detail in Supplementary Section 4.7). For genetic perturbations, we found that multi-hot embeddings of the Reactome database [33] worked the best. Reactome is an open-source, open-access, manually curated and peer-reviewed pathway database. Essentially, for each perturbed gene, we created a multi-hot vector depending on whether it belongs to a certain Reactome pathway or not. We then trained a CatBoost to predict the expected response to a perturbation based on Reactome embeddings.

- **Geneformer:** We used the pretrained Geneformer model published at `https://huggingface.co/ctheodoris/Geneformer` (downloaded in August 2023). We either fine-tuned the model according to their respective instructions or used their embeddings with a CatBoost model, then chose the approach that performed better. The source Geneformer model was pre-trained on a large corpus of unperturbed pooled single-cell gene expression datasets. We note that Geneformer is limited to processing at most 2048 transcripts, we therefore created multiple references that were independently fed to Geneformer to produce gene embeddings for all perturbed genes across the evaluated datasets. Geneformer is described in detail in [26].

- **scGPT:** We used the pretrained whole-human scGPT model published at `https://github.com/bowang-lab/scGPT` (downloaded in August 2023). We either fine-tuned the model according to their respective instructions or used their embeddings with a CatBoost model, then chose the approach that performed better. scGPT is trained on pooled single-cell gene expression data not under perturbations. scGPT is described in detail in [27].

- **GenePT:** We followed the strategy outlined in Chen and Zou [29] to generate gene embeddings from the National Center for Biotechnology Information (NCBI) summary text descriptions of genes using ChatGPT 3.5, and then used those gene embeddings as the input data from which a Catboost model was trained to make downstream predictions for evaluation. GenePT is described in detail in [29].

- **GEARS:** The model covered in Supplementary Section 4.7 and in detail described in [11].

	Learning rate	Learning rate decay	MLP layers	Dropout rate	Hidden dim.	Embed. dim.	Batch size
Grid search	0.001	0.99	1	0	256	128	1000
	0.002	0.97	2	0.1	512	64	5000
	0.01			0.25		32	10000
Target context							
Replogle (K562) CRISPRi	0.002	0.99	2	0	512	32	5000
Replogle (RPE1) CRISPRi	0.005	0.99	2	0	512	32	5000
LINCS (HT29) Compounds	0.002	0.97	2	0.1	256	128	1000
LINCS (HELA) Compounds	0.002	0.97	2	0.1	256	128	1000
LINCS (HA1E) Compounds	0.002	0.97	2	0.1	256	128	1000
LINCS (MCF7) CRISPR-KO	0.002	0.97	2	0.1	256	128	1000
LINCS (HT29) CRISPR-KO	0.002	0.97	2	0.1	256	128	1000
LINCS (A549) CRISPR-KO	0.002	0.97	2	0.1	256	128	1000
Training contexts							
Replogle all	0.002	0.99	2	0	512	32	5000
LINCS all	0.002	0.97	2	0.1	256	128	1000

Supplementary Table 2 **Hyper-parameter selection.** The table consists of three parts. In the first part shown are the hyper-parameters that were used in the grid search as a part of the model selection. The second part shows the selected hyper-parameters for the post-perturbation transcriptome prediction benchmark. The third part shows hyper-parameters used for the embedding-related tasks, where two LPMs were trained on all LINCS and Replogle data.

- **CPA:** The model covered in Supplementary Section 4.7 and in detail described in [15].

Apart from weighted Adam [67] optimizer and early stopping (patience of 10 epochs) that were fixed throughout our study, the rest of the hyper-parameters were tuned as indicated in Supplementary Table 2. The baselines were given the same computational budget and were tuned according to the recommendations from the corresponding publications. It took about 50 epochs for all training runs to early stop based on the validation performance on the validation set. Early stopping used RMSE as the stopping criterion.

Embedding evaluation. For embeddings-related experiments, we used consensus hyper-parameter configuration from our predictive performance experiments. The perturbation embeddings from the trained models were first used to assess the information content by classifying molecular functions associated with the perturbations. For that purpose, we trained CatBoost models with 5 different random seeds to classify perturbations into functional mechanisms as annotated by Replogle et al. [9] based on the perturbed genes' mechanisms using LPM embeddings and various state-of-the-art gene embeddings (Reactome, STRING, Achilles, Gene2vec, scGPT, Geneformer and GenePT) (Figure 3). We then compared the ability of the compared embeddings to accurately assign functional mechanisms to genes using the Area under the Curve (AUC) using the unweighted mean in a one-vs-rest multi-class computation to aggregate across the functional categories. To evaluate how well the trained LPM can identify mechanisms of action of the compound inhibitors, we used drug repurposing hub data (`https://repo-hub.broadinstitute.org/repurposing`). We kept only targets and compounds that appear in our data (from Supplementary Table 1), and only information about compounds that inhibit/suppress the targets (removing compounds with non-inhibiting mechanisms).

Gene-gene network inference evaluation. To learn gene-gene interaction networks, we used the state-of-the-art method proposed by Deng and Guan [58] (Guanlab) complemented with data imputed by an LPM. Guanlab is generally biased towards predicting edges for

the nodes that have been perturbed in the training data, meaning that almost no edges are predicted from non-perturbed nodes. This impacts the recovery rate of causal gene interactions (as measured by the FOR) [68]. To remedy this and to leverage the LPM perturbation predictions, we implemented a two-step approach. The first step involved running the Guanlab algorithm on the original training data, and taking the top 2500 predicted edges. We then retrained a Guanlab model, this time only on the prediction from the LPM, taking the top 2500 predicted edges. The final output consisted of the union of those two sets of 2500 edges. Compared to the original Guanlab (top 5k) model without imputation, we obtained a significantly lower FOR for the same number of output edges, indicating that leveraging LPMs allowed recovering many more genetic interactions.

In silico perturbation study of predicted *PKD1* upregulators. We trained an LPM on pooled chemical and genetic perturbation data from LINCS and, after training, evaluated the set of PRC queries (P = all 5 310 chemical perturbations in LINCS, R = all 978 L1000 transcripts, C = HA1E LINCS). Because *PKD1* was not in the set of landmark transcripts directly experimentally measured in LINCS, we additionally trained a Catboost [31] regressor model (hyperparameters: 512 iterations, 100 round early stopping patience) using HA1E cells to infer the *PKD1* value corresponding to the L1000 transcripts predicted by LPM. This produced an in silico predicted *PKD1* value for each of the 5 310 chemical perturbation included in LINCS. We then ranked the chemical perturbations from highest predicted *PKD1* upregulation to lowest and filtered for clinical stage drugs. We removed natural products and early stage investigational drugs from the ranking because it is a priori unclear whether their safety profile or off-target effects may impede clinical utility as potential ADPKD therapeutics. We note that the ability of LPM to perform context-specific predictions was essential for the study results as predictions in other cellular model systems did not surface the same chemical perturbations as top regulators of *PKD1* - indicating a dependence of the results of the in silico perturbation experiment on the biological context in kidney cells.

Retrospective matched cohort study to evaluate simvastatin in ADPKD. To validate the predicted *PKD1* upregulator simvastatin in real-world clinical data, we performed a matched cohort study using retrospective data collected in the Optum© de-identified Electronic Health Record database collected from a decentralised network of healthcare provider organisations, including 2 000 hospitals, 7 000 clinics and 100 million unique individuals, from 2007 to 2023 in the United States (US). Among those clinically diagnosed with ADPKD (International Classification of Diseases [ICD] 10: Q61.2 or Q61.3 [69]; ICD 9: 753.12 or 753.13), we created two cohorts for comparison: a treatment cohort consisting of individuals that were exposed to Simvastatin for more than one year (date of first and last prescription greater than 365 days apart) and a "not exposed" cohort of individuals that did not receive any of the statins (simvastatin, fluvastatin, pitavastatin, cerivastatin, mevastatin) predicted by a LPM to upregulate *PKD1.* For the treatment cohort, we used 1 year after their first recorded simvastatin prescription as the index date, that is, there was a 1 year induction period for simvastatin to influence cystogenesis. For the "not exposed" cohort, we picked an index date between their first and last entry into the EHR at random (uniform distribution) in line with the target trial emulation framework [70]. Censoring was applied on the date of the last recorded entry for an individual in the EHR and the first recorded entry in the EHR was considered the first date of observation for each individual. We applied 1:1 propensity score matching (PSM) [71] using a non-linear XGBoost [72] propensity score estimator with a 1% calliper to ensure the two cohorts are comparable based on their covariates as measured prior to their respective index dates (see Supplementary Table 3 for matching covariates). This yielded two cohorts comparable in their observed covariates (Supplementary Table 3) for which we used a Nelson-Aalen estimator [73] using the `lifelines` package [74] to produce

Property	Before matching Simvastatin	No statin use	After matching Simvastatin	No statin use
Patients	1 716	48 555	1 594	1 594
ADPKD	100%	100%	100%	100%
Age	63.75	52.41	63.40	64.72
95% CI	(41.10, 79.58)	(14.33, 79.84)	(41.34, 79.77)	(42.34, 79.21)
Index year	2012.94	2016.73	2013.23	2012.42
95% CI	(2008.85, 2018.49)	(2010.31, 2021.83)	(2009.12, 2018.64)	(2008.85, 2017.90)
Sex (Female)	42.31%	52.08%	43.41%	42.10%
Ethnicity				
Caucasian	85.02%	73.95%	84.32%	85.57%
African American	9.91%	12.40%	10.54%	9.66%
Other/Unknown	3.55%	11.39%	3.64%	3.14%
Asian	1.52%	2.26%	1.51%	1.63%
Hispanic	3.26%	6.56%	3.14%	3.01%
Region				
Region Midwest	61.83%	41.94%	61.98%	66.50%
Region South	13.05%	23.98%	13.93%	9.60%
Region Northeast	11.83%	15.21%	11.04%	10.92%
Region West	10.31%	14.06%	10.10%	10.54%
Region Other/Unknown	2.97%	4.82%	2.95%	2.45%
Type 1 diabetes	4.66%	1.87%	3.70%	5.02%
Type 2 diabetes	42.60%	19.04%	40.09%	45.29%
Hypertension	94.52%	65.20%	94.17%	95.73%
Cardiovascular disease	56.00%	28.21%	54.02%	59.47%
Obesity	43.88%	23.65%	41.84%	46.68%
Proteinuria	27.10%	11.53%	24.47%	28.48%

Supplementary Table 3 **Retrospective study cohort descriptions.** Cohort descriptions for the retrospective matched cohort study conducted to evaluate impact of Simvastatin on progression to ESRD among ADPKD diagnosed individuals (Figure 5). Each row corresponds to a covariate measured across the cohorts (Simvastatin 1 year+ exposure vs no statin use) at index date before and after (the two rightmost columns) propensity score matching. The cohorts included in the study are well balanced and comparable across the indicated covariates that include unspecific (for example, age) and specific (for example, diabetes and obesity) risk factors for ESRD.

cumulative hazard estimates for progression to ESRD (ICD 10: N18.* or N17 or N19 or Z99.2; ICD 9: 585.* or 586 or 584.9 or V45.11 [75]; * indicates inclusion of the primary code and all sub-codes). We chose the Nelson-Aalen estimator because it does not require the proportional hazards assumption. We note that we were not able to validate other predicted *PKD1* upregulators in real-world clinical data because there are not enough individuals that received the other predicted medications and are diagnosed for ADPKD in the Optum© EHR database. We note that the observed association of long-term simvastatin exposure in reducing ESRD progression in ADPKD diagnosed individuals does not conclusively prove that *PKD1* is the responsible mechanism as it is possible that simvastatin reduces ESRD events through other or multiple means.

Tolvaptan. Tolvaptan is the only medicine currently approved for treatment of ADPKD [76]. Tolvaptan is an *AVPR2* antagonist that reportedly slows disease progression by inhibiting production of cyclic adenosine 3',5'-monophosphate (cAMP) [77], and therefore a different mechanism than the mechanism targeting *PKD1* upregulation that we investigated in the presented in silico study. Because the training data used to develop LPM did not contain reference data for tolvaptan perturbations, we predicted the effects of its analogue perturbation in silico via CRISPRi of *AVPR2*. We found that the predicted impact of inhibiting *AVPR2* on *PKD1* was (although also directionally positive) minor compared to statins predicted to upregulate *PKD1* (Figure 5) - indicating that tolvaptan indeed likely acts through a different mechanism than *PKD1* upregulation.

Extended related work

In silico perturbation modeling, in the sense of predicting post-perturbation outcome, has been studied intensively in recent years. A traditional approach is to first infer a perturbation screen-specific biological network and then use the inferred network to predict perturbation response with respect to outcomes of interest [10, 12, 78]. For example, CellOracle [10] infers a linear representation of a gene regulatory network (GRN) which is then used to predict gene expression and cell identity changes caused by hypothetical perturbations of transcription factors (TFs). This approach is more classical in the sense that it involves several complex steps (clustering, graph inference, prediction, dimensionality reduction), as opposed to deep learning-based approaches where models are trained "end-to-end". Other non-end-to-end approaches include mechanistic modeling of cell machinery via ODEs [12, 79] or using linear regression to describe perturbation-readout relationships [80]. Most of the above-mentioned methods are also limited in the type of perturbations they model (for example, mutations of TFs) or the readouts they predict (for example, cell viability or growth rate). In contrast, LPM handles arbitrary types of perturbations or readouts. A notable advantage of traditional approaches is that they offer a certain degree of interpretability such as an explicit representation of a GRN.

More recently, deep learning methods have been popularized [81] due to their ability to model functions of arbitrary complexity and leverage massive amounts of data from high-throughput screens. Variational autoencoder (VAE)-based methods [13–16, 19, 64] autoencode readouts, normally transcriptome, through a latent space in which perturbations are modeled and applied. VAE-based methods are rather flexible in terms of the types of perturbations and covariates they can deal with. For example, CPA [15] incorporates drug dosage information and handles combinatorial perturbations such as multi-gene knockouts. CPA and related methods learn perturbation and covariate embedding spaces end-to-end, in a fully data-driven fashion. One disadvantage of VAE methods is that they require each perturbation to be experimentally perturbed before the effect of perturbing the combination can be predicted, that is, they are limited to *in-vocabulary* settings. Like in LPM, this could be alleviated through data pooling across different studies, however, due to the rigid encoder-decoder architecture, VAEs are generally not suitable for handling heterogeneous screens that may differ in terms of data format and dimensions. Data pooling can be enabled using complex tricks that involve architectural changes that limit the size of the pool [14].

To enable *out-of-vocabulary* prediction, a line of methods that incorporate prior knowledge emerged [11, 14, 16]. ChemCPA [14] uses molecular structures to construct drug embeddings and predicts cellular response to drugs that do not appear in the experimental data. GEARS [11] uses publicly available Gene Ontology (GO) and gene co-expression data to construct gene embeddings to extrapolate response prediction to unseen genetic perturbations. However, predefined representations of perturbations have their own issues. Firstly, the extrapolation power is limited by the coverage of those perturbations. For example, it is unclear how to obtain common perturbation joint embedding spaces of pharmacological and genetic perturbations, which could be very useful in identifying drug-gene relationships as shown in Figure 3. Secondly, unlike representations learned end-to-end, predefined ones are created independently of the perturbation response prediction task hence may not be sufficiently informative of functional relationships among perturbations. Another disadvantage of GEARS [11] is that is not suitable for multi-context settings.

Our work follows an end-to-end learning but is focus on integrating perturbation screens from different contexts. To our knowledge, ChemCPA [14] is the only method from the VAE space which attempts to deal with heterogeneity in multi-context settings. ChemCPA uses what they call "architecture surgery", wrapping pre-trained VAE with supplementary encoders and decoders to adapt the model to the target data format. This strategy unfortunately (i) ignores the absence of missing readouts in the pre-training data; (ii) is applicable only when

all the pre-training data is of equivalent format, otherwise, undesirable subsetting of readouts must be applied which discards relevant experimental information. In contrast to ChemCPA, the proposed non-autoencoder architecture of an LPM can be trained on datasets that contain arbitrary types of perturbations, readouts, and contexts, without making assumptions about the dimensionality of the data. Another major difference between VAE-based approaches such as CPA and LPM is the structure that such methods impose on the latent space. For example, CPA takes a so-called "information removal" strategy which optimizes auxiliary loss functions to minimize the mutual information among embeddings encouraging them to be independent. This step is required due to the fact that the encoder can tangle the information content of the embeddings. However, LPM is encoder-free and thus does not need to explicitly disentangle information from the output of the encoder. We note that LPM achieves disentangled embeddings without explicitly optimizing for it (Figure 3).

A critical component of LPM and related works is the representation of perturbations in the form of embeddings. LPM and CPA train embeddings from scratch, learning them from data in an unbiased fashion. On the other hand, GEARS and the Catboost baseline (from our benchmarks) utilise predefined embeddings. GEARS embeddings are based on Gene Ontology (GO) [82] and gene co-expression knowledge graph on top of which a graph neural network is applied. For the Catboost baseline, we tried multiple different ways to represent both readout and perturbation embeddings, including Gene2vec [34], multi-hot encoded Reactome (`https://reactome.org`), and STRING embeddings [32].

Foundational models are the relatively new line of work based on Transformer neural network architecture [83]. Transformer-based architectures are used to learn gene embeddings from abundant observational gene expression data using various strategies for tokenising transcript counts. These models (frequently referred to as "foundation models") include Geneformer [26], scGPT [27], scFoundation [28], and GenePT [29]. Unlike LPM, Transformer-based methods are trained on observational data and hence lack the data to make transportable causal inferences, and furthermore produce only gene embeddings that cannot directly be used to understand pharmacological perturbations. Note also that it is not established whether the tokenisation required by Transformer-based models leads to performance improvements over more straightforward representations of tabular experimental transcriptomics data. The contrast between foundational models and LPM has been in more detailed covered in the introduction of this paper.

Worth-noting are a sequence of methods that have a causal perspective to perturbation modeling. CINEMA-OT [84] and Cell-OT [17] use optimal transport to infer treatment effects and predict responses of single cells to genetic or drug-based perturbations, with a particular focus on pairing (matching) intrinsically unpaired distributions of perturbed and non-perturbed cells. CINEMA-OT attempts to explicitly separate confounding sources of variation from perturbation effects. The advantage of these methods is their ability to predict post-perturbation outcome for single cells (individual treatment-effect analysis). sVAE+ [81] is a variant of the sVAE with a Bayesian aproach for learning sparse mechanism shifts. sVAE+ models perturbations in single-cell space as unknown but sparse interventions on latent space, using concepts of sparsity to enhance its utility. sVAE+ offers interpretability features and covers novel aspects such as latent units recovery and causal target identification. These methods are not designed for learning from multi-context data.

Understanding the generalizability of perturbation models necessitates examining their capabilities in handling in-vocabulary (IV) and out-of-vocabulary (OOV) predictions. LPM, as presented here, operates entirely within an IV framework, meaning they can predict only combinations of symbols encountered during training. While this may seem limiting, it's important to note: *(i)* The number of perturbation studies is steadily increasing, suggesting that the symbolic space will soon be substantially covered; *(ii)* There's potential to replace randomly initialized embeddings with predefined ones, similar to approaches like GEARS, to

facilitate OOV predictions for perturbations, contexts, or readouts. Variational Autoencoder (VAE)-based approaches are also predominantly IV. However, unlike LPM, and with the exception of models like ChemCPA, they often struggle with heterogeneity in multi-context settings. GEARS can predict effects of OOV perturbations, limited to symbols present in the Gene Ontology, but it cannot perform OOV context predictions. Foundational models like scGPT are capable of IV perturbation prediction and OOV context prediction. In summary, while LPM is currently restricted to IV predictions, the expanding dataset landscape and potential integration of predefined embeddings offer ways to enhance their OOV capabilities.

Other results

Extended results from the benchmarks presented in the main part of the paper. The detailed predictive performance benchmark is given in Supplementary Figure 1. For the task of gene regulatory network inference comparison, we present the full performance statistics for the gene regulatory network inference task outlined in Supplementary Table 4.

		K562 cells			
Model	Mean Rank	Wasserstein Rank	FOR Rank	Wasserstein Distance	FOR
Mean Difference (top 5k)	3.0	4	2	0.371 ± 0.005	0.158 ± 0.004
**LPM+Guanlab**	4.0	7	1	0.318 ± 0.005	0.150 ± 0.005
Guanlab (top 1k)	4.0	2	6	0.430 ± 0.010	0.181 ± 0.001
Mean Difference (top 1k)	4.0	1	7	0.523 ± 0.007	0.182 ± 0.003
Guanlab (top 5k)	4.5	6	3	0.327 ± 0.005	0.161 ± 0.002
Moebius	5.0	5	5	0.341 ± 0.002	0.176 ± 0.003
Betterboost	5.5	3	8	0.420 ± 0.006	0.186 ± 0.001
NoPerturb+Guanlab	6.0	8	4	0.266 ± 0.004	0.161 ± 0.004

		RPE1 cells			
Model	Mean Rank	Wasserstein Rank	FOR Rank	Wasserstein Distance	FOR
Moebius	3.5	4	3	0.293 ± 0.003	0.115 ± 0.004
**LPM+Guanlab**	4.0	7	1	0.274 ± 0.006	0.087 ± 0.008
Guanlab (top 1k)	4.0	2	6	0.496 ± 0.008	0.126 ± 0.003
Mean Difference (top 5k)	4.5	5	4	0.289 ± 0.004	0.116 ± 0.005
Mean Difference (top 1k)	4.5	1	8	0.556 ± 0.009	0.142 ± 0.003
NoPerturb+Guanlab	5.0	8	2	0.260 ± 0.009	0.091 ± 0.008
Betterboost	5.0	3	7	0.474 ± 0.011	0.132 ± 0.004
Guanlab (top 5k)	5.5	6	5	0.276 ± 0.004	0.119 ± 0.004

Supplementary Table 4 Complete gene network inference performances on the K562 (top) and RPE1 (bottom) cell lines with 50% of interventional data.

Additional results on post-perturbation prediction. We conducted an additional experiment on the effects of CRISPRi genetic perturbations on cell viability, based on the study by [35], which explored how genetic interactions impact viability. This dataset includes three cell viability-related readouts derived from pairwise perturbations across two cell types,

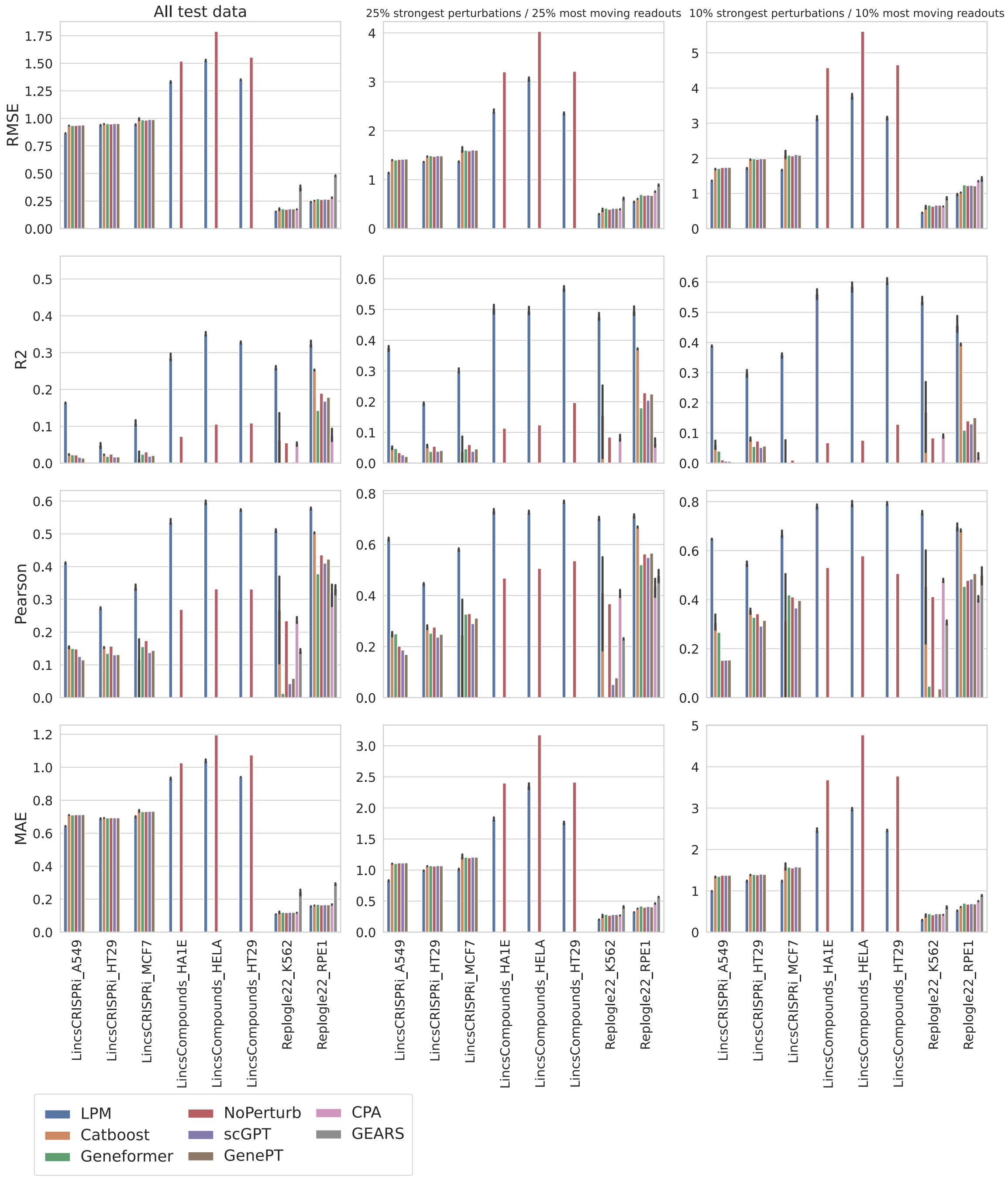

Supplementary Fig. 1 | Detailed performance comparison in predicting unperformed experiments. LPM consistently performs better across considered datasets, metrics, and with respect to different portions of the test set in predicting outcomes of unobserved perturbations. Given that the transfer learning setting studied here consists of only one dataset, we expect performance could potentially improve as we include more datasets in the training process.

demonstrating the adaptability of our method to data beyond transcriptomics and in low-dimensional settings. Our experimental setup followed the approach described above: each single perturbation involved in a pairwise combination was included during training, while the specific combinations themselves were withheld. Note that because we used only the average of two replicates, GEARS was not applicable in this case, as it currently supports only single-cell data. Similarly, scGPT could not be applied due to the non-transcriptomic nature of this dataset. Results are shown in Supplementary Figure 2.

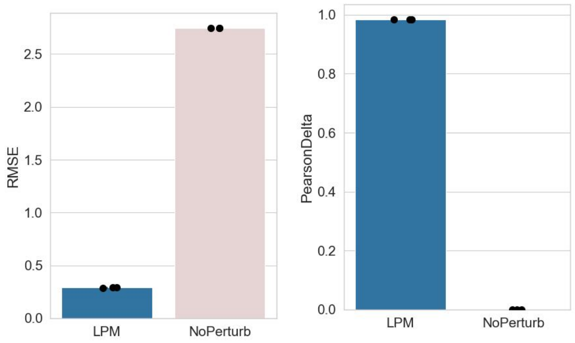

Supplementary Fig. 2 | Predicting unperformed experiments on perturbation screens that measure cell viability [35]. LPM shows considerable performance improvements in comparison to the simple NoPerturb baseline.
